# Case Report of an Acute Complex Perilunate Fracture Dislocation Treated with a Three-Corner Fusion

**DOI:** 10.1155/2018/8397638

**Published:** 2018-05-23

**Authors:** Graeme Matthewson, Samuel Larrivee, Tod Clark

**Affiliations:** ^1^University of Manitoba, S013-750 Bannatyne Avenue, Winnipeg, MB, Canada R3E 0W2; ^2^Pan Am Clinic Foundation, 75 Poseidon Bay, Winnipeg, MB, Canada R3M 3E4

## Abstract

Perilunate fracture dislocations are a rare but devastating injury, which is often missed on initial presentation leading to significant delays in treatment. With the delay in treatment and a high energy mechanism of injury, patients are at increased risk of developing complex regional pain syndrome following trauma. In this report, we review the case of a 57-year-old left-hand dominant female who presented to a clinic with a five-and-a-half-week-old transtriquetral, perilunate fracture dislocation with comminution of the scaphoid facet. Due to the increased likelihood of a secondary procedure and low probability of a satisfactory outcome with open reduction internal fixation secondary to the loss of the scaphoid articulation, a salvage procedure was deemed her best option. To our knowledge, this is the first case reported in the literature in which a scaphoidectomy, triquetromy, and midcarpal fusion (three-corner fusion) was performed in the acute setting for a perilunate fracture dislocation.

## 1. Introduction

Perilunate injuries are relatively rare, accounting for only 7% of pathologies to the carpus. However, a staggering number of these injuries are not diagnosed, with up to 25% of perilunate dislocations being missed on initial presentation [1–3]. The importance of identifying these injuries is highlighted by the significant complications produced by missed or improperly treated injuries including median nerve injury, chronic carpal instability, avascular necrosis of the mal-reduced lunate, complex regional pain syndrome, unreliable return of function, and posttraumatic arthrosis requiring a secondary procedure [2–5]. In addition to patient factors, surgical complications are increased due to extensive fibrosis and scarring and the inability to achieve an anatomic reduction of the carpal bone complex [4]. Here, we present a case of a 57-year-old female who suffered a complex perilunate fracture dislocation treated by scaphoidectomy, triquetromy, and midcarpal fusion (three-corner fusion). To our knowledge, this is the first reported case in the literature for the treatment of an acute perilunate dislocation with this procedure.

## 2. Case Report

**Figure 1:**
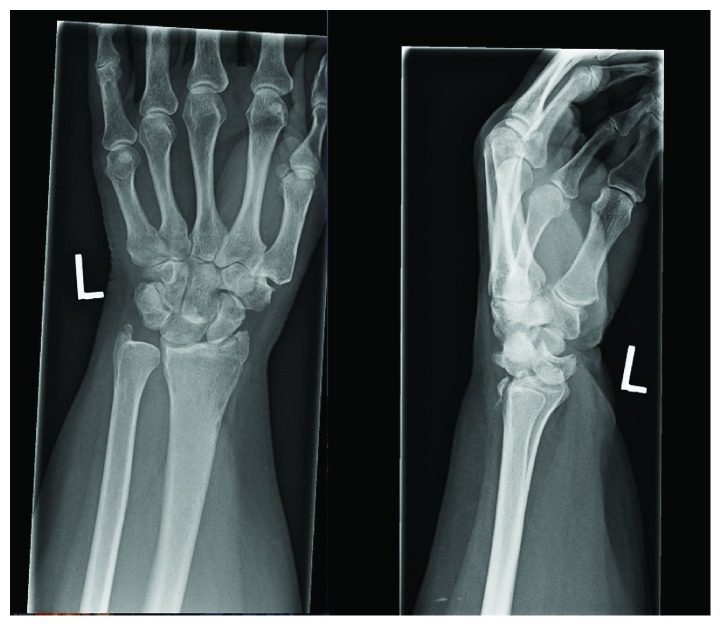
AP and lateral images of the initial radiographs taken in the peripheral hospital. Note the comminuted scaphoid facet, subtle triquetral fracture (AP) as well as the obvious perilunate dislocation (lateral).

A 57-year-old left-hand dominant woman presented to the arthroplasty clinic for a consultation regarding her right knee osteoarthritis. On her visit, the consulting surgeon noticed an improperly placed below elbow cast which was well past her metacarpal phalangeal joints, greatly restricting movement of her left fingers. She was previously seen at a peripheral hospital approximately five weeks prior, after sustaining a fall on an outstretched hand (FOOSH) from standing height. Noting this, the consulting surgeon ordered an X-ray which revealed that the patient had a missed perilunate dislocation with a fracture of the triquetrum and a comminuted impaction fracture of the scaphoid facet (Figure 1). This prompted the surgeon to put in an immediate call to the senior author for an immediate consult.

**Figure 2:**
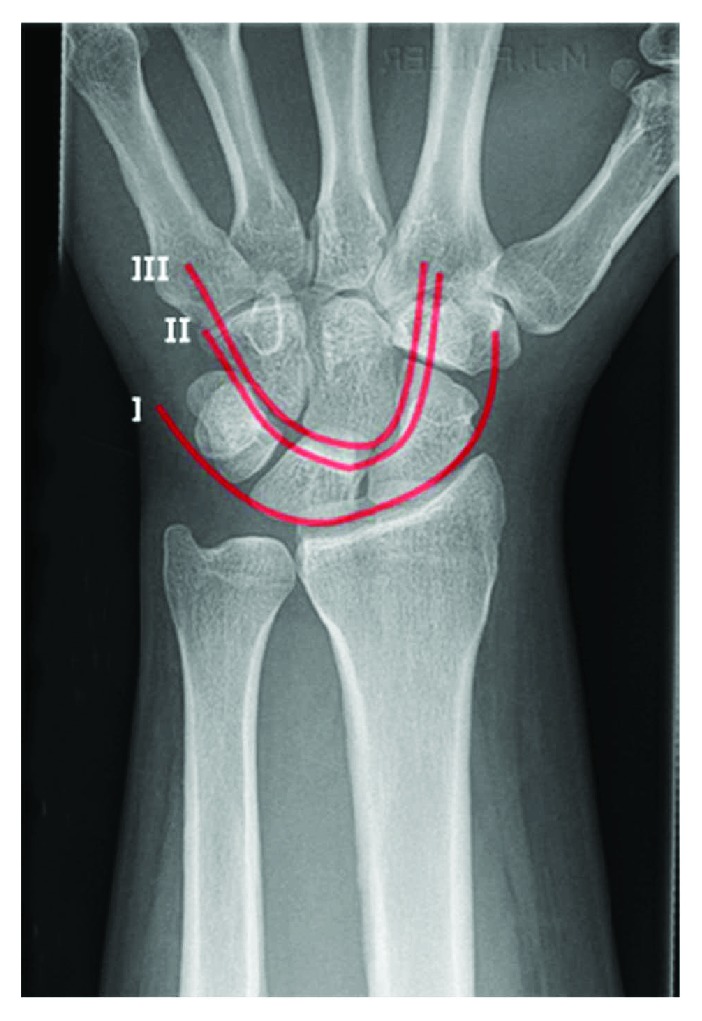
Radiograph demonstrating Gilula's lines. Line I represents the proximal articular surfaces of the proximal carpal row. Line II represents the distal articular surfaces of the first carpal row. Line III represents the proximal articular surfaces of the distal carpal row. Disruption of these lines is indicative of a boney (greater arc) or ligamentous (lesser arc) injury.

**Figure 3:**
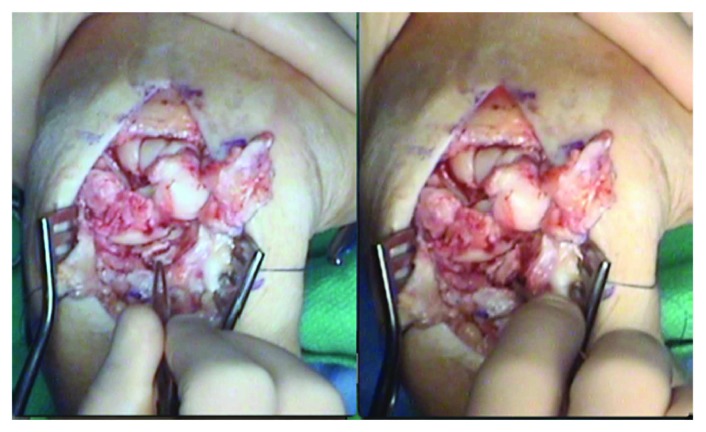
Examination of the comminuted scaphoid facet. (a) Obvious impaction injury. (b) Impaction fragment loose and nonadherent has a high likelihood of secondary arthrosis.

In the clinic, the patient complained of considerable volar wrist pain with occasional numbness and tingling to the hand. Examination of the hand revealed significant swelling and hyperalgesia of the skin of the wrist and hand. She was otherwise neurovascularly intact to all nerve distributions. X-ray examination included an anteroposterior (AP) and lateral imaging of the left hand and wrist. On the anterior radiograph, there was an obvious disruption of Gilula's lines (see Figure 2 for comparison), as well as an impaction injury to the scaphoid facet (Figure 3), and fracture of the triquetrum. On the lateral X-ray, there was an obvious dorsal dislocation of the lunocapitate joint consistent with a perilunate fracture dislocation. Treatment options were discussed with the patient regarding open reduction internal fixation (ORIF) compared with a salvage procedure. Since the scaphoid facet was badly injured, the decision to perform an ORIF versus a salvage procedure was left until the status of the cartilage could be determined. A proximal row carpectomy and a three-corner fusion were discussed as salvage procedures in the acute setting. The patient agreed with the treatment plan, was placed into a well-fitting cast, and was booked for surgery for the following week.

**Figure 4:**
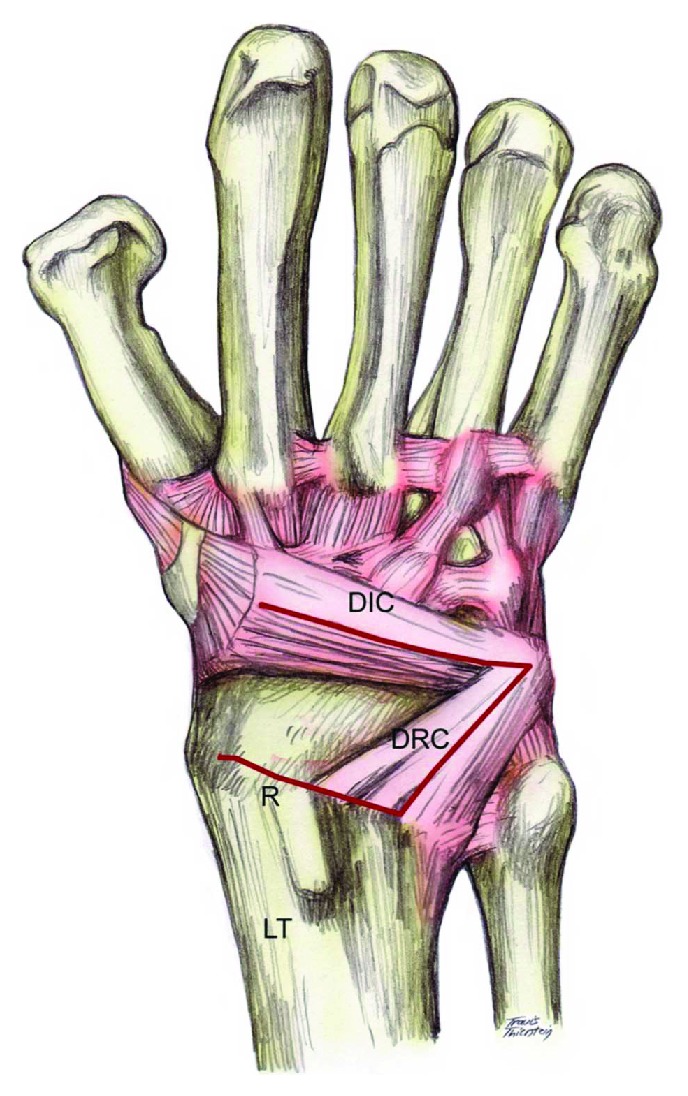
Illustration of the capsular incision used in the ligamentous sparing Berger capsulotomy. Note the radially based flap created. DRC: dorsal radiocarpal ligament. DIC: dorsal intercarpal ligament. The image was reprinted with a permission from Medscape Drugs & Diseases (https://emedicine.medscape.com/) and Perilunate Fracture Dislocations, 2017, available at https://emedicine.medscape.com/article/1240108-overview.

**Figure 5:**
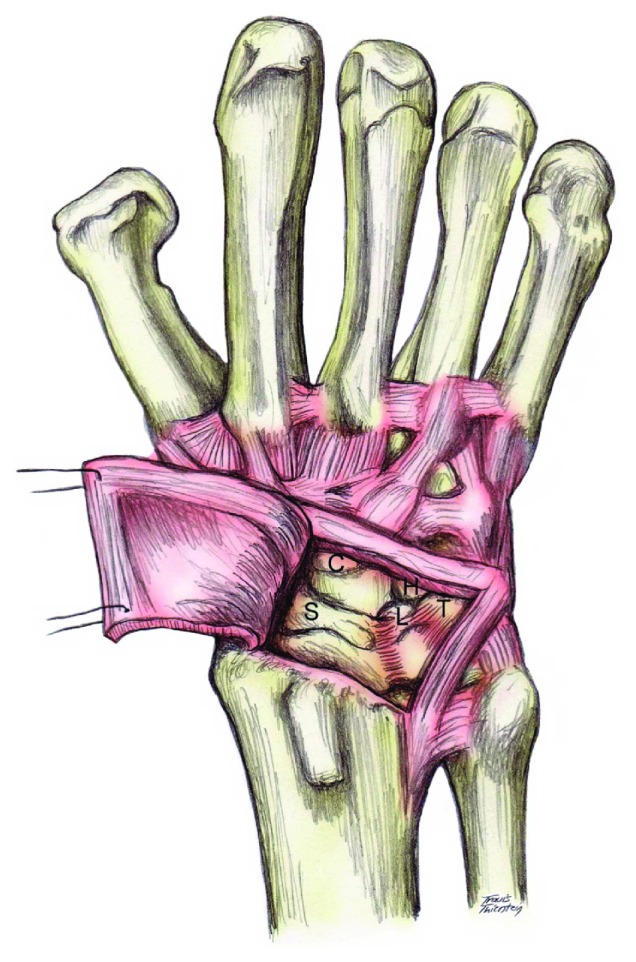
Visualization of the carpal bones once the radially based flap is lifted. S: scaphoid; C: capitate; L: lunate; T: triquetrum; H: hamate. The image was reprinted with permission from Medscape Drugs & Diseases (https://emedicine.medscape.com/) and Perilunate Fracture Dislocations, 2017, available at https://emedicine.medscape.com/article/1240108-overview.

**Figure 6:**
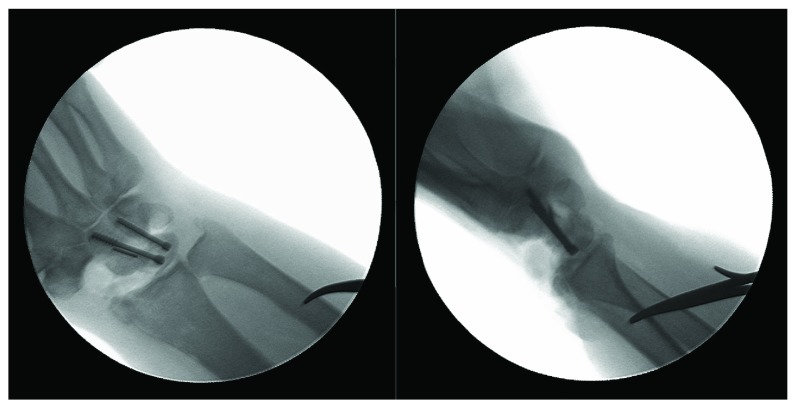
Intraoperative fluoroscopic images confirming screw position and proper alignment of the midcarpal fusion.

The patient was brought to the operating room and was placed supine with the use of an arm board. The patient was prepped and draped in usual fashion, and an operative time out was performed. A dorsal approach to the wrist was used, utilizing a Berger ligamentous sparing capsulotomy (Figures 4 and 5). Once the carpus was exposed, it was evident that the second carpal row was dislocated dorsal to the lunate. Examination of the articular surfaces of the carpus revealed a healthy appearing lunate and lunate fossa with a significantly comminuted and impacted scaphoid facet. In addition, there was significant damage to the proximal capitate chondral surface. As the proximal capitate articulation is key for optimal results in a proximal row carpectomy and with the significant chondral damage and comminution of the scaphoid facet, the decision was made to perform a three-corner fusion. The cartilage was removed from the distal aspect of the lunate and proximal aspects of the capitate and hamate with a scalpel, and the articular surfaces were contoured using a 3 mm burr with copious irrigation to prevent thermal necrosis. K-wires were placed across the lunocapitate joint, with the position confirmed on fluoroscopy, and a 3 mm headless compression screw was used to affix the joint. This was followed by a lunohamate screw placed in a similar fashion. The final screw position was confirmed on imaging (Figure 6), followed by irrigation and closure of the wound. The patient was then placed in a volar slab and sent to recovery in a stable condition. Immobilization was maintained for a total of 6 weeks. Upon follow up, the patient's clinical course deteriorated with the development of postoperative complex regional pain syndrome. On the most recent follow up appointment at 12 weeks postoperatively, the patient began to have some relief of her symptoms following the implementation of an extensive physiotherapy and pain control protocol.

## 3. Discussion

The treatments of acute (defined as <6 weeks) [6] and chronic perilunate dislocations are considerably varied. Currently, most clinicians would opt to perform an ORIF in the acute and many times chronic setting. This would involve either the reconstruction of the scapholunate ligament or, if possible, primary repair, in addition to the pinning of the lunotriquetral joint and fixation of any fractures that occurred. However, particularly in the chronic setting, ORIF has had unreliable outcomes and high rates of secondary arthrosis with some authors reporting rates as high as 50–100% [3, 7]. With a mean incidence of 38%, a staggering number of perilunate injuries go on to develop posttraumatic arthrosis. In a retrospective review, Forli et al. [8] found in patients who suffered a perilunate injury that 11/18 cases had progressed to develop signs of posttraumatic degenerative changes with a minimum of 10 years postsurgery, and some authors have reported the appearance of degenerative changes as early as few years from injury [9]. Of the patients showing radiographic change, 2 had excellent results, 2 had good results, 5 had fair results, and 2 had poor results according to the Mayo Wrist Score (MWS). Following this, Breyer et al. [10] retrospectively reviewed 37 patients who sustained either perilunate dislocation or perilunate fracture dislocation treated with ORIF at an average of 4 years. They found similar results with an excellent result in one patient, good results in 7 patients, fair results in 18 patients, and poor results in 11 according to the MWS. As referenced by Forli et al. [8], at this point, the literature fails to show a clear correlation between posttraumatic arthrosis and functional outcomes. This was corroborated by Kailu et al. [11], where they found that satisfactory results could be obtained up to 25 weeks from the time of injury. However, they also noted that the patients with substantial chondral damage had poor results, regardless of the timing of surgery. This led the authors to recommend a salvage procedure in the setting of significant cartilage damage. Due to the number of less than optimal results with ORIF, many authors have opted for salvage procedures as the primary operation, particularly in the setting of severe carpal trauma [12]. In a retrospective cohort analysis, Muller et al. [1] compared ORIF against proximal row carpectomy (PRC) for the treatment of acute perilunate dislocations. In their study, 13 patients were treated by ORIF while 8 patients were treated by PRC with an average follow up of 35 months. The results of this study showed that ORIF and PRC were quite comparable in the acute setting, with shorter surgical time and postoperative immobilization. With significant cartilage damage and comminution of the scaphoid facet, it was felt that the maintenance of the radioscaphoid joint would result in significant morbidity leading the surgeon to opt for a salvage procedure. Due to the damage to the capitate articular cartilage, the results from a PRC would have been considerably compromised in our patient. With the additional fracture to the triquetrum, the senior author decided to perform a three-corner fusion as an alternative to four-corner fusion, which has shown equivalent results, only with additional ROM in ulnar deviation [13]. Unfortunately, the patient went on to develop complex regional pain syndrome (CRPS). CRPS is a devastating and relatively uncommon condition with a predilection towards patients with injuries to the hand or wrist. While researchers have not clearly defined the precise cause of CRPS, they have been able to identify many of the contributing factors, including inflammation, central sensitization, sympathetic mediation, cortical reorganization, and neurogenic inflammation [5]. As all of these states are triggered and exacerbated by an untreated injury, delay in diagnosis for hand and wrist injuries inevitably increases the chances of developing this condition. Unfortunately, due to the significant ligamentous injury and fractures of the distal radius and triquetrum, in combination with the delay in diagnosis, our patient was at a significant risk of developing CRPS. This made performing only one procedure even more important with regard to long-term outcomes as to not expose the patient to a secondary procedure, risking a second insult, and subsequent episode of CRPS.

## 4. Conclusion

In summary, with the high rate of secondary pathology and an increased risk of complications with multiple procedures, performing a salvage surgery in the acute setting for severe perilunate fracture dislocations could prevent a patient from undergoing a secondary surgery. This case highlights the importance of prompt diagnosis of perilunate injuries, the negative consequences associated with missed injuries, and an alternative primary procedure to the traditional ORIF of perilunate fracture dislocations.
